# Renaming the Jornal of Brasileiro de Pneumologia (JBP) to Respiratory Research and Clinical Practice (RRCP)

**DOI:** 10.36416/3086-3910/e20260097

**Published:** 2026-03-05

**Authors:** Marcia Margaret Menezes Pizzichin

**Affiliations:** 1 Editor in Chief

## DEAR COLLEAGUES

We are thrilled to announce that the Jornal Brasileiro de Pneumologia will be renamed to Respiratory Research & Clinical Practice. The rapidly evolving landscape of scientific publishing necessitates periodic re-evaluation of journals and, at times, their branding. Since its establishment in 1975 as the Journal of Pneumology (JP), it has served as the official publication of the Brazilian Society of Pneumology and Phthisiology (SBPT), The name was updated to Brazilian Journal of Pneumology (JBP) in 2003. 

In the following 23 years, the JBP progressively consolidated its position as a reliable journal known for its robust and rigorous peer review process, scientific credibility, and commitment to ethical and transparent publishing practices. However, the submissions of original articles were mainly published by respected Brazilians scientists, perhaps biased by the name of the journal that suggests the focus of publications predominantly oriented by regional issues. Despite of these difficulties in 2024, the JBP achieved a notable milestone by ranking in the Q2 quadrant among 105 international scientific journals in Respiratory Medicine, securing the 42nd position, a journal impact factor of 3.0, and an H-index of 52. Our citation index, as reported by Scopus, currently stands at 4.0, underscoring the relevance of our publications over the past five years, with 78% of original articles cited. In 2026 the JBP was elevate from B1 to A2 By the ranking national and international scientific journal of the National Council for Research (Qualis-CNPq).

We take enormous pride in the journal’s achievements, but the question that remains is “can we do better” in the coming years”? Given the emerging trends and the necessity for greater visibility, renaming our journal to Respiratory Research & Clinical Practice (RRCP), aims to remove the regional connotations of our current journal JBP. This change has the potential to enhance our international recognition and better align our work with the global research landscape in respiratory medicine.

The idea of changing in the name of JBP originated in a 2025 meeting of the SBPT Board of Directors and has progressed through discussions involving of Associated Editors and former Chief Editors of the JBP to further explore the impact of this initiative. In 2026, with the support of the SBPT Board of Directors, and of the majority of the JBP Associated Editors the change of the name of JBP was approved. Although we considered several excellent names, many already had ISSN and were therefore unsuitable. Ultimately, we settled on the name Respiratory Research & Clinical Practice (RRCP) and were successfully granted an ISSN.

## 1. REASONS FOR THE NAME CHANGE

Renaming our journal to Respiratory Research & Clinical Practice presents several significant advantages. Primarily, the inclusion of the term “Respiratory” clearly communicates our focus and establishes a vital connection with the global research community in this field. This connection is expected to promote increased submissions from a wider pool of national and international authors, encourage interdisciplinary collaboration, and broaden our readership base. These are critical factors in establishing a prominent presence in the global scientific arena. Moreover, the new name accurately reflects the journal’s dual emphasis on original research and clinical practice. This clarity is likely to enhance the quality of translational research articles, enriching our content and making it more relevant to both researchers and clinicians.

Additionally, an updated name can serve as a catalyst for growth, improving our journal’s impact factor and indexing status over time. By modernizing our brand and targeting a broader audience, we enhance our potential for higher-quality submissions and citations, which are essential for maintaining our journal’s standing and relevance in the field.

## 2. POTENTIAL DOWNSIDES

While we are enthusiastic about the potential benefits of this transition, we also acknowledge the challenges it may present. In the first three years following the name change, we anticipate that metrics such as the Citation Index, Impact Factor, and Qualis CNPq may be affected due to the necessary transition between the existing name and the new one. This fragmentation could temporarily impact our perceived standing in academic circles but is to be expected during the process of rebranding.

To address this challenge, we plan to implement several strategies, including robust marketing campaigns to reestablish our brand identity and encourage consistent submission patterns. During this transitional phase, it is essential to maintain a steady flow of publications. Author engagement will be critical to our success, and we kindly ask our current contributors, both Brazilian and international, to continue submitting their work to ensure that the journal retains its momentum and continues to flourish throughout the rebranding process. By fostering ongoing author participation, we can develop a stronger, uninterrupted pipeline of high-quality research, ultimately benefiting the journal and our community.

In summary, renaming the Jornal Brasileiro de Pneumologia (JBP) to Respiratory Research & Clinical Practice (RRCP) represents a significant step toward greater internationalization and relevance in the field of respiratory medicine. We believe that, with continued support, hard work, and contributions from our authors, we can navigate this transition successfully and elevate our journal’s impact. Thank you for your engagement and commitment to this important initiative.


Marcia M. M. Pizzichini Editor in Chief 


